# Determinants of Mental Illness Among Humanitarian Migrants: Longitudinal Analysis of Findings From the First Three Waves of a Large Cohort Study

**DOI:** 10.3389/fpsyt.2019.00545

**Published:** 2019-08-02

**Authors:** Sam Cooper, Joanne C. Enticott, Frances Shawyer, Graham Meadows

**Affiliations:** ^1^Southern Synergy, Department of Psychiatry, Monash University, Melbourne, VIC, Australia; ^2^Department of General Practice, Southern Synergy, Department of Psychiatry, Monash University, Melbourne, VIC, Australia; ^3^Centre for Mental Health, Melbourne School of Population and Global Health, University of Melbourne, Melbourne, Australia; ^4^Mental Health Program, Monash Health, Melbourne, VIC, Australia

**Keywords:** refugee, humanitarian migrants, mental health, longitudinal study, social support

## Abstract

**Background:** As refugee numbers grow worldwide, understanding prevalence and determinants of mental illness in this population becomes increasingly important.

**Methods:** We used longitudinal data to examine the initial years of resettlement in Australian refugees with a focus on ethnic-like social support. Three annual waves from a longitudinal, nationally representative cohort of 2,399 humanitarian migrants recently resettled in Australia were examined for two mental illness outcomes: post-traumatic stress disorder indicated by positive PTSD-8 screen and “high risk of severe mental illness” (HR-SMI) by Kessler Psychological Distress Scale (K6) ≥19. Generalized linear mixed models examined demographic and resettlement factors.

**Findings:** Contrary to predictions, high prevalence of positive screens for mental illness persisted over 3 years. At baseline, 30.3% (95% CI, 28.5–32.2) screened positive for post-traumatic stress disorder (PTSD), and 15.4% (95% CI, 14.0–16.9) had HR-SMI. Over the 3 years, 52.2% met screening criteria for mental illness. PTSD was associated with older age, females, Middle Eastern birthplace, increasing traumatic events, more financial hardships, having a chronic health condition, and poor self-rated health. HR-SMI was associated with females, Middle Eastern birthplace, unstable housing, more financial hardships, having a chronic health condition, poor self-rated health, and discrimination. Also contrary to predictions, like-ethnic social support was positively associated with PTSD (OR, 1.51; 95% CI, 1.10–2.09).

**Interpretation:** There is high prevalence of positive screens for mental illness throughout initial years of resettlement for refugees migrating to Australia. Our unexpected finding regarding like-ethnic social support raises future avenues for research. Predictors of mental illness in the post-migration context represent tangible opportunities for intervention and are likely relevant to similar resettlement settings globally.

## Introduction

The number of forcibly displaced people worldwide has currently reached levels second only to the number shortly after World War II (1). The United Nations High Commissioner for Refugees (UNHCR) estimates that there were 22.5 million refugees and 2.8 million asylum seekers worldwide at the end of 2016, equating to, on average, 20 people displaced from their homes every minute (1). Australia’s humanitarian intake represents the third highest rate of UNHCR humanitarian resettlement worldwide, behind the United States and Canada, and is committed to a programme in 2018–2019 aiming to permanently resettle 18,750 humanitarian migrants (1, 2). Individuals acquiring visas prior to arrival in Australia are considered as offshore humanitarian migrants and individuals acquiring visas after arriving in Australia as onshore humanitarian migrants (2). The Australian offshore humanitarian migrant programme resettles individuals under a Permanent Protection visa. The Australian onshore humanitarian migrant programme resettles individuals under the “Refugee” or “Special Humanitarian Programme” visa categories (2). The onshore and offshore migrant groups are herein described as “humanitarian migrants” for this study.

Humanitarian migrants appear to be at a greater risk of mental illness, especially post-traumatic stress disorder (PTSD), when compared with wider host and non-refugee migrant populations (3–5). Authors of a meta-analysis considering conflict exposed populations and refugees showed prevalence of PTSD as 30.6% (95% CI, 26.3–35.6%) and depression as 30.8% (95% CI, 26.3–35.6%) (6). Forced migration involves major transitions in cultural and socioeconomic systems and the reconstruction of social networks (7). Additionally, the stresses of premigration trauma, intra-migration voyages, and socioeconomic instability in the post-migration context may contribute to the high prevalence of mental illness found in refugee populations (6, 8, 9). While the predictors of mental illness may appear similar to non-refugee populations, it is likely they obtain extra significance in the refugee context. This may be true for social support, which appears beneficial for mental health, yet may be dependent on the source of the support received. Schweitzer et al. (10) found only like-ethnic social support was significantly negatively associated with PTSD, whereas support received from the wider host population was not. These results are similar to other studies, both quantitative (11) and qualitative (12, 13), which suggest a greater benefit of like-ethnic support when compared with outside sources of support. Conversely, other studies provide evidence that longer time spent with like-ethnic community confers a greater risk for mental illness (14). Understanding the predictors of mental illness is essential for directing interventions in the post-migration context, as mental illness has been shown to negatively impact many aspects of resettlement.

Additionally, prevalence of mental illness throughout the initial resettlement period seems high among humanitarian migrants (15). Over the long term, the prevalence of mental illness appears determined by the degree of trauma exposure and post-migration socioeconomic context (16). However, whether this prevalence declines (11, 17) or persists (18) over the course of early resettlement is unclear, particularly in the Australian context.

The Building a New Life in Australia (BNLA) study is a 5-year longitudinal, population-level cohort study of recently arrived humanitarian migrants to Australia. Past analysis of the BNLA survey has demonstrated a high prevalence of severe mental health problems in Australian humanitarian migrant populations and has shown cross-sectional associations between post-migration factors and mental illness (15, 19). Another published study examined the relationships between PTSD, refugees’ parenting styles, and their children’s mental health using the three waves of the BNLA study (20).

Studying refugee populations has proven difficult given the inherent cultural and linguistic diversity of these “hidden” populations, making them difficult to engage at a population level (21). Past studies considering the prevalence, course, and predictors of mental illness in humanitarian migrant populations have been limited by cross-sectional design, opportunistic sampling techniques, and small sample sizes. Additionally, the bulk of the previous literature does not reflect the current distribution of nationalities seen displaced globally today. As a result, the conclusions that can be drawn from previous nonrepresentative cohorts and cross-sectional data are limited.

Our study considered data from the largest, nationally representative, longitudinal cohort survey of refugee populations to date: the Building a New Life in Australia survey (22). We examined the first three annual waves of data, which encompass the initial 3 years of permanent resettlement in an Australian humanitarian migrant population. Our study aimed to explore the prevalence, course, and predictors of mental illness in this population through time. The study was exploratory in nature but had two *a priori* hypotheses: firstly, prevalence of mental illness would decrease over time, and secondly, the presence of like-ethnic social support would be negatively associated with mental illness.

## Methods

### Data Source

We used the first three annual waves of the BNLA study, a nationally representative, longitudinal cohort study examining the first 5 years of resettlement in a humanitarian migrant population (22). The population studied were onshore and offshore humanitarian migrants who first settled in Australia from May to October 2013. The first annual wave occurred between October 2013 and March 2014, the second between October 2014 and February 2015, and the third between October 2015 and February 2016.

The initial study was funded by the Australian Government (Department of Social Services) and coordinated by the Australian Institute of Family Studies (AIFS). All potentially identifying details from survey responses were deemed confidential to maintain the anonymity of respondents. Further descriptions of the BNLA study design can be found in previously published analysis of wave 1 (15) and in publicly available documents (19, 22).

### Participants

A census approach was used to sample participants from the Australian Department of Border Protection and Immigration (DIBP) settlement database, which contained demographic and contact details for all individuals granted Australian humanitarian migrant visas during the selected study dates.

Eligible study participants fulfilled the inclusion criteria:

- Over 15 years old
- Onshore humanitarian migrant who was granted a permanent residence visa between May and December 2013 (6 months before fieldwork dates of BNLA study)
  or
  Offshore humanitarian migrant that arrived in Australia between May and December 2013 (6 months before fieldwork dates of BNLA study)
- Living in 1 of 11 deemed-confidential study locations around Australia
- Written consent given to participate in study

Principal applicants (PA) are the primary adults listed on the visa application and were the initial individuals contacted for participation and were considered as the lead participants for the study. Invitations to participate were sent *via* mail, followed by a phone call and subsequent home visits by researchers (22). Secondary applicants (SAs) are other members of the migrating unit noted on the PA visa application, for example, a spouse or child. If a PA was recruited for the study, invitations to participate in study were extended to SAs. This migrating unit was considered as the primary sampling unit for the study. Individuals aged 15 years and above were included in the study in order to maximize the available data points with the view that individuals at age 15 years in wave 1 would be 18+ over the course of the study. We were able to examine the effect of age by including age variables in our multivariate models.

### Outcome Measures

Questionnaires were administered to participants using multiple methods, to accommodate responses from individuals who had poor reading literacy (19, 22). Waves 1 and 3 were mostly administered *via* computer-assisted self-interview, whereas wave 2 was mostly computer-assisted telephone interview. Interpreters and interviewers were available for both methods upon participant request (22). Given the diversity of national and ethnic groups within the cohort, the written questionnaire was translated into 14 languages for wave 1 and with the availability of interpreters; 19 languages were covered in total for the study (22).

Two dichotomous primary outcome measures were examined in this study: “High Risk of Severe Mental Illness (HR-SMI)” as measured by the Kessler-6 (K6) (23) and “PTSD” as measured by the PTSD-8 (24). Cronbach’s alpha was used to calculate the internal consistency of the K6 and PTSD-8 in our study. Predictor variables are described in **Supplementary Appendices**.

The K6 is a short-form version of the widely used Kessler-10 (23). The scale is used to indicate likelihood of severe mental illness (23). The K6 scores ranged from 9 to 30, and a score ≥19 was used as a cutoff as screening positive for HR-SMI (22). In our study, internal consistency of the K6 in wave 1 was 0.93.

The PTSD-8 is a screening questionnaire that assesses the three symptom clusters in the Diagnostic and Statistical Manual of Mental Disorders, version IV (DSM-IV) diagnosis for PTSD (24). It is derived from the Harvard Trauma Questionnaire, which has shown good performance in cross-cultural populations (25). Participants with responses of *“Sometimes”* or *“Most of the time”* in at least one of each of the PTSD symptom clusters were considered as screening positive for PTSD. In our study, internal consistency of the PTSD-8 in wave 1 was 0.96.

A secondary aggregate outcome variable of “any mental illness” was included if either HR-SMI or PTSD was present at a given wave. As this aggregate outcome did not present any new information, it is not presented in results.

### Statistical Analyses

We used Stata/SE 14.2 for Windows for all analyses (26). Population sample weights provided by the AIFS were used for all analyses, unless otherwise stated (22). Sociodemographic characteristics of the baseline cohort were summarized. Categorical data were described using frequency, percentage, and 95% confidence intervals (CIs). Continuous data were described using median and IQR, as data were not normally distributed. The prevalence of outcome variables at the population level were represented graphically with 95% CIs and significance of prevalence change across waves assessed using binomial tests.

A variable selection process with four stages (see *Appendix*) was used to identify the final independent variables used in the final generalized linear mixed models (GLMM). Firstly, univariate ordinal regressions were used to examine associations between baseline candidate variables and primary outcome variables, with variables retained at *p* < 0.1. Second, correlated variables were examined using Pearson’s correlation coefficients, and one candidate variable was selected from two or more variables when *r* > 0.5. Selection was influenced on clinical and statistical relevance as well as amount of missing data. This step was conducted by two authors (SC and JE) so that highly correlated variables were removed from the final models. Third, we analyzed the outcomes in the panel data using GLMM *via xtlogit* in stata (26) (as dichotomous outcomes) with migrating (family) unit and individuals specified as random effects and a variable denoting “wave” as fixed. Variables at *p* < 0.1 and time and social support variables were retained for a second iteration of the logit model. Social support and time variables were then only retained at *p* < 0.1 in a third iteration of the model. Variables relating to social support and time were retained until this stage regardless of their significance, as they were directly related to the hypotheses. In the last variable selection stage, candidate variables were modeled using *xtlogit* over 1,000 bootstrap samples at 95% resampling of the original data set. The number of times each candidate variable achieved significance (*p* < 0.05) was recorded. Candidate variables were selected for use as independent variables in final models if they were identified as significant in 1,000 bootstraps over 50% of the time. The results of this variable selection process are shown in the **Supplementary Appendix**.

The final models examined associations between the outcomes and independent variables selected in the four-stage process previously discussed, with migrating unit and individuals specified as random and other variables as fixed effects in the GLMM (27). To ensure robustness, our final models are based on 200 bootstrap samples drawn with 95% resampling. A sensitivity analysis was run by removing those variables with more than 10% missing data across all three waves and comparing the results with those of the final model. Final “Akaike’s information criteria” (AIC) and “Bayesian information criteria” (BIC) were used to compare the relative “goodness of fit” for each model (28).

Forest plots were used to graphically represent significant findings from final models.

### Missing Data

A missing data analysis was done to evaluate the potential effects of response bias. To do this, we examined potential associations between missing data and outcome measures over three waves using logistic regression. Demographic variables examined were age, gender, region or birth, and trauma. There were no significant associations between demographic variables and nonresponse to K6 or PTSD-8 over all three waves, including nonresponse due to attrition. This supported the assumption that the data appeared to be missing at random and no imputation for missing values was done.

### Ethics Approval

The original BNLA study was approved by the Australian Institute of Family Studies ethics committee, which is registered with the National Health and Medical Research Council. Names of participants and potentially identifying information were withheld from the data source, meaning no individual can be identified by researchers.

Ethics exemption for secondary analysis of the data was granted by Monash University Human Research Ethics Committee.

## Results

### Participants

**Figure 1:**
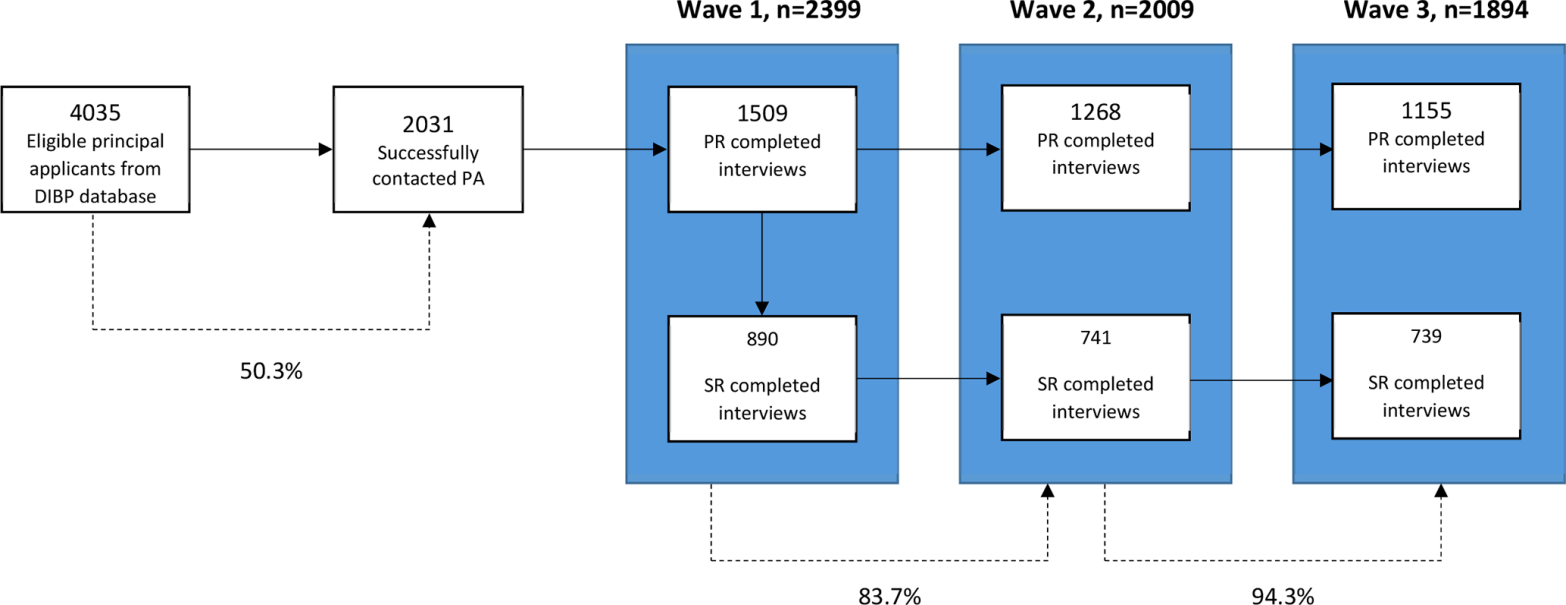
Flowchart of participants. Sampling process resulting in baseline interview completion rates of principal and secondary applicants. Author’s own figure. These sample counts are unweighted. DIBP, Department of Immigration and Border Protection. PA, principal applicants. PR, principal respondents. SR, secondary respondents. % = represents percentage participants retained between stages.

There were 1,509 principal respondents and 890 secondary respondents who completed interviews at baseline (**Figure 1**) (19, 22). A total of 2,399 completed interviews at baseline, 2,009 at wave 2, and 1,894 at wave 3. The overall response rate between baseline to wave 3 was 79.0% (**Table 1**).

**Table 1:** Three-wave participation and retention rates of the Building a New Life in Australia (BNLA) survey. These sample counts are unweighted.

	Wave		
	*1*	*2*	*3*
*Interviews completed (n)*	2,399	2,009	1,894
*Retention rate of wave 1 (%)*	100.0	83.7	79.0
*Wave response rate from previous wave (%)*	–	83.7	94.3

– = not calculated.

### Demographics

**Table 2** shows that the majority of baseline participants were male (54.0%), originating from the Middle East (44.8%), married or partnered (57.4%), and unemployed (92.4%) with 6–12 years of pre-arrival schooling (48.8%).

**Table 2:** Demographics of wave 1 (baseline) cohort. These sample counts are unweighted. Median age calculated as data were not normally distributed.

Variable	Response	All participants
**Age, median (IQR)**		24 (13–39)
**Gender, *n* (%)**	*Male*	1,307 (54.5)
	*Female*	1,092 (45.5)
**Region of birth, *n* (%)**	*Middle East*	1,270 (52.9)
	*Central Asia*	611 (25.5)
	*Southern Asia*	218 (9.1)
	*Africa*	157 (6.5)
	*South-East Asia*	137 (5.7)
	*Missing data*	6 (0.3)
**Married or partnered, *n* (%)**	*Yes*	1,376 (57.4)
	*No*	888 (37.0)
	*Missing data*	135 (5.6)
**Employed, *n* (%)**	*No*	2,230 (93.0)
	*Yes*	145 (6.0)
	*Missing data*	24 (1.0)
**Education level pre-**	*Never attended school*	380 (15.8)
**arrival, *n* (%)**	*6 or less years of school*	473 (19.7)
	*6–12 years of school*	1,137 (47.4)
	*Trade/technical qualification*	143 (6.0)
	*University degree*	243 (10.1)
	*Missing data*	23 (1.0)
**SEIFA Quintile of Relative Socioeconomic Disadvantage 2011**	*1–2*	1,571 (65.4)
	*3–4*	383 (15.9)
	*5–6*	229 (9.6)
	*7–8*	161 (6.7)
	*9–10*	55 (2.3)
**Total, *n* (%)`**		2,399 (100)

Onshore and offshore migrants differed significantly by gender (*p* < 0.001), region of birth (*p* < 0.001), employment (*p* < 0.0001), pre-arrival education (*p* < 0.0001), and time since arrival (*p* < 0.0001).

### Social Support

**Table 3** shows that approximately half of the baseline participants had received at least some support from their “national or ethnic” community (51.3%) and religious community (46.9%); a smaller proportion had received at least some support from other community groups (33.5%). A substantial minority of participants had received no support from ethnic, religious, or other community groups (41.4%).

**Table 3:** Sources of support received by participants over time. These sample counts are unweighted.

		Wave		
Variable	Response	1	2	3
**Received like-ethnic support, *n* (%)**	Yes	766 (31.93)	619 (30.81)	516 (27.24)
	Sometimes	434 (18.09)	427 (21.25)	408 (21.54)
	No	1,068 (44.55)	891 (44.35)	912 (48.15)
	*Missing	131 (5.46)	72 (3.58)	58 (3.06)
**Received religious support, *n* (%)**	Yes	657 (27.39)	501 (24.94)	510 (26.93)
	Sometimes	397 (16.55)	370 (18.42)	369 (19.48)
	No	1,172 (48.85)	1,067 (53.11)	952 (50.26)
	*Missing	173 (7.21)	71 (3.53)	63 (3.33)
**Received other community support, *n* (%)**	Yes	392 (16.34)	406 (20.21)	327 (17.27)
	Sometimes	361 (15.05	373 (18.57)	376 (19.85)
	No	1,459 (60.82)	1,138 (56.65)	1,104 (58.29)
	*Missing	187 (7.79)	92 (4.58)	87 (4.59)
**Degree of support, *n* (%)**	High	844 (39.13) 39.13)	744 (39.64)	728 (40.67)
	Low	1,313 (60.87)	1,133 (60.36)	1,062 (59.33)
	*Missing	242 (10.00)	131 (6.52)	104 (5.49)
**Received no support, *n* (%)**	Yes	883 (36.81)	633 (31.51)	768 (40.55)
	No	1,516 (63.19)	1,376 (68.49)	1,126 (59.45)
**Total**		2,399 (100)	2,009 (100)	1,894 (100)

*Missing indicates nonresponse to question but does not include missing data due to attrition.

### Prevalence of Mental Illness

**Figure 2:**
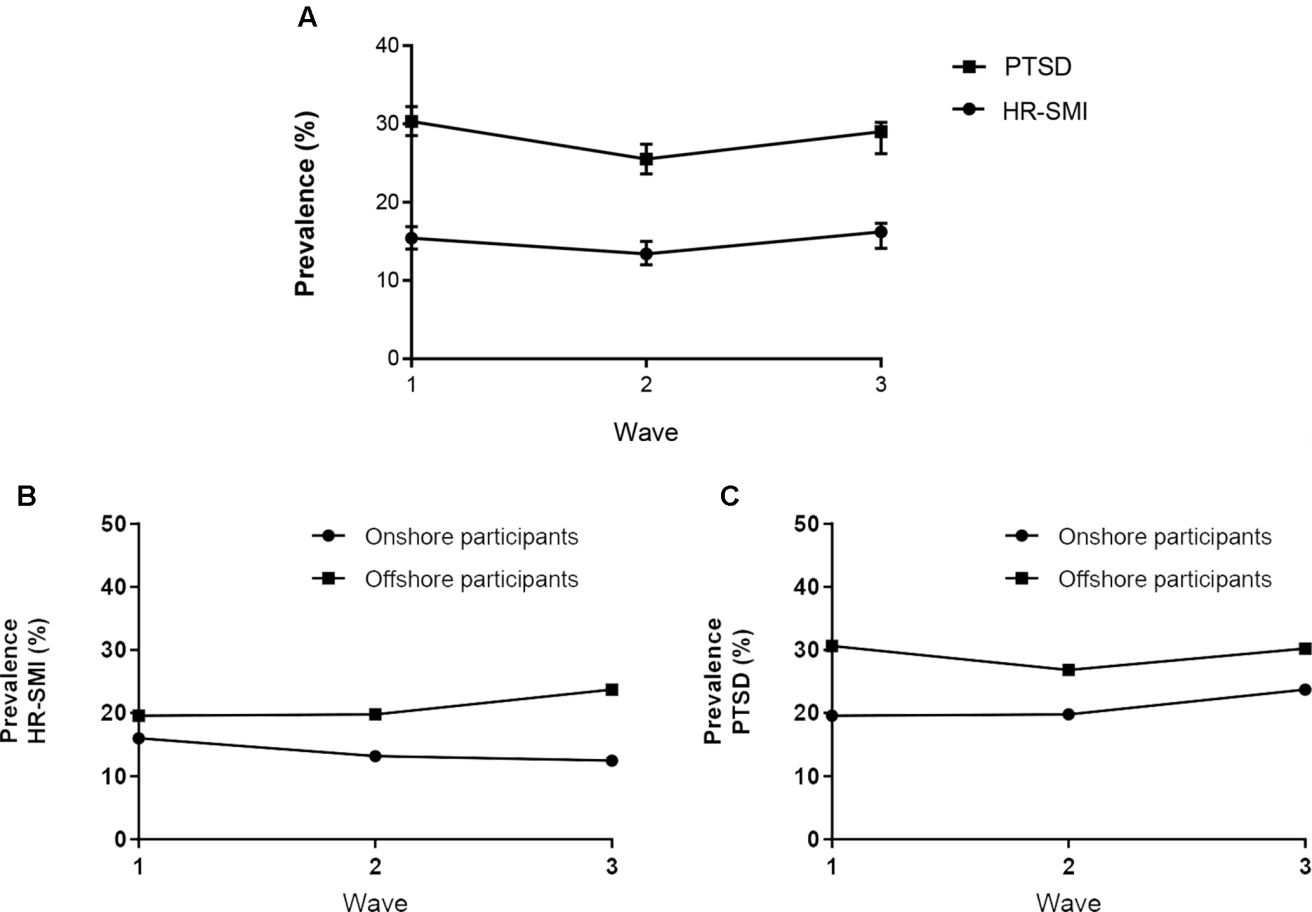
Prevalence of mental illness over time. **(A)** PTSD, post-traumatic stress disorder and; HR-SMI, high risk of severe mental illness. **(B)** HR-SMI for onshore and offshore participants. **(C)** PTSD or onshore and offshore participants.

At baseline, 30.3% (95% CI, 28.5–32.2) of participants screened positive for PTSD, and 15.4% (95% CI, 14.0–16.9) of participants screened positive for HR-SMI (see **Figure 2** and **Table 4**). The majority of the total study participants (52.2%) screened positive for at least one of PTSD or HR-SMI at some point over the three waves (see **Table 5**). Only 32.3% of wave 3 participants were free from mental illness at all waves of the study.

**Table 4:** Prevalence of mental illness measures at each wave. These prevalence estimates are weighted using population weights for each waves.

		Wave 1			Wave 2			Wave 3		
Variable		*n*	%	*CI*	*n*	%	*CI*	*n*	%	*CI*
**HR-SMI**	*Yes*	370	15.4	(14.0–16.9)	270	13.4	(12.0–15.0)	296	16.2	(14.1–17.3)
	*No*	1,946	81.1	(79.5–82.6)	1,734	86.3	(84.7–87.7)	1,494	82.6	(77.0–80.7)
	*Missing*	83	3.5	(2.8–4.3)	5	0.26	(0.1–0.6)	43	2.3	(1.7–3.2)
**PTSD **	*Yes*	727	30.3	(28.5–32.2)	512	25.5	(23.6–27.4)	533	29.1	(26.2–30.2)
	*No*	1,548	64.5	(62.6–66.4)	1,450	72.2	(70.2–74.1)	1,234	67.3	(63.0–67.3)
	*Missing*	123	5.1	(4.3–6.1)	46	2.3	(1.7–3.1)	66	3.6	(2.8–4.6)
**Any mental illness**	*Yes*	830	34.6	(32.7–36.5)	582	29.0	(27.0–31.0)	629	33.2	(31.1–35.4)
	*No*	1,455	60.7	(58.7–62.6)	1,388	69.1	(67.0–71.1)	1,142	62.3	(58.1–62.5)
	*Missing*	114	4.8	(4.0–5.7)	39	1.9	(1.4–2.6)	62	3.4	(2.6–4.3)
**Prescribed medication for emotional health**	*Yes*	334	13.9	(12.6–15.4)	185	9.2	(0.8–0.11)	–	–	–
	*No*	2,046	85.3	(83.8–86.6)	1,093	54.4	(52.2–56.6)	–	–	–
	*Missing*	19	0.8	(0.5–0.1)	731	36.4	(34.3–38.5)	–	–	–
**Received professional help**	*Yes*	–	–	–	–	–	–	639	34.9	(32.7–37.1)
**for emotional problems**	*No*	–	–	–	–	–	–	1,153	62.9	(60.7–65.1)
	*Missing*	–	–	–	–	–	–	40	2.21	(1.6–3.0)

– = missing data as question not asked at wave. CI, Agresti and Coull 95% confidence intervals; PTSD, post-traumatic stress disorder; HR-SMI, high risk of severe mental illness.

**Table 5:** Incidence and 3-year prevalence of mental illness. These sample counts are weighted using population weights for each wave.

	Weighted incidence							
	*Wave 1*		*Wave 2*		*Wave 3*		*3-year prevalence*	
Variable	*n*	*%*	*n*	*%*	*n*	*%*	*n*	*%*
***PTSD***	727	30.3	228	11.4	145	7.7	1100	45.9
***HR-SMI***	370	15.4	146	7.27	111	5.9	627	26.1
***At least 1 primary outcome at any wave***	–	–	–	–	–	–	1,254	52.2
***No primary outcomes at all waves***	611	25.5	611	30.4	611	32.3	611	25.5
**Total**	2,399	100	2,009	100	1,894	100	2,399	100

– = not calculated. PTSD, post-traumatic stress disorder; HR-SMI, high risk of severe mental illness.

Between waves 1 and 2, there was a significant decrease in the prevalence of PTSD and severe mental illness (*p* = 0.001). There was no significant overall decrease between waves 1 and 3 for severe mental illness; however, for PTSD, there was (*p* = 0.021) (see **Supplementary Appendix**).

### GLMM Results

**Figure 3:**
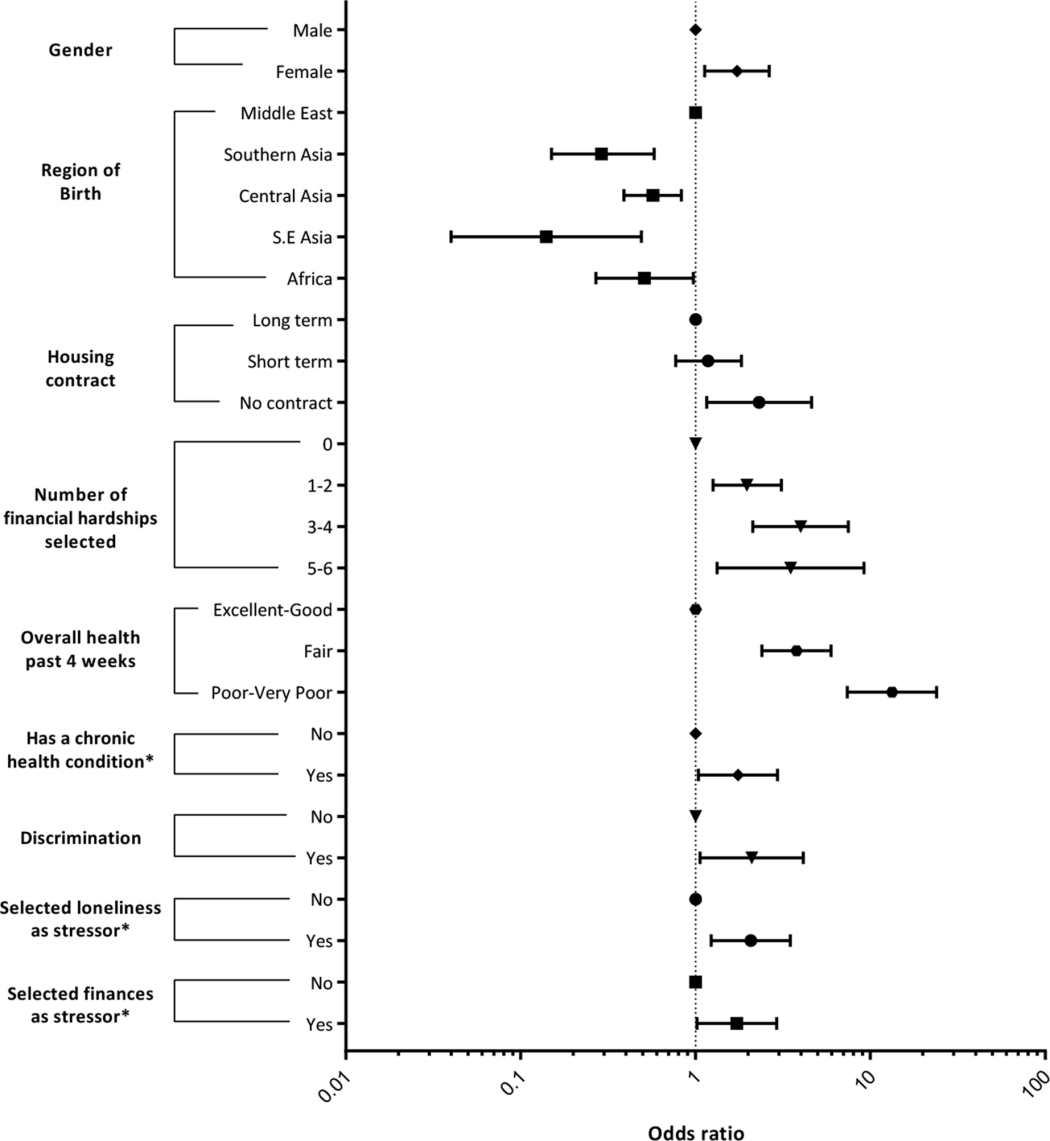
Forest plot significant predictors of high risk of severe mental illness. Summary of GLMM applied to the 3 annual waves in the BNLA data set. GLMM, generalized linear mixed model; BNLA, Building a New Life in Australia study.

**Figure 4:**
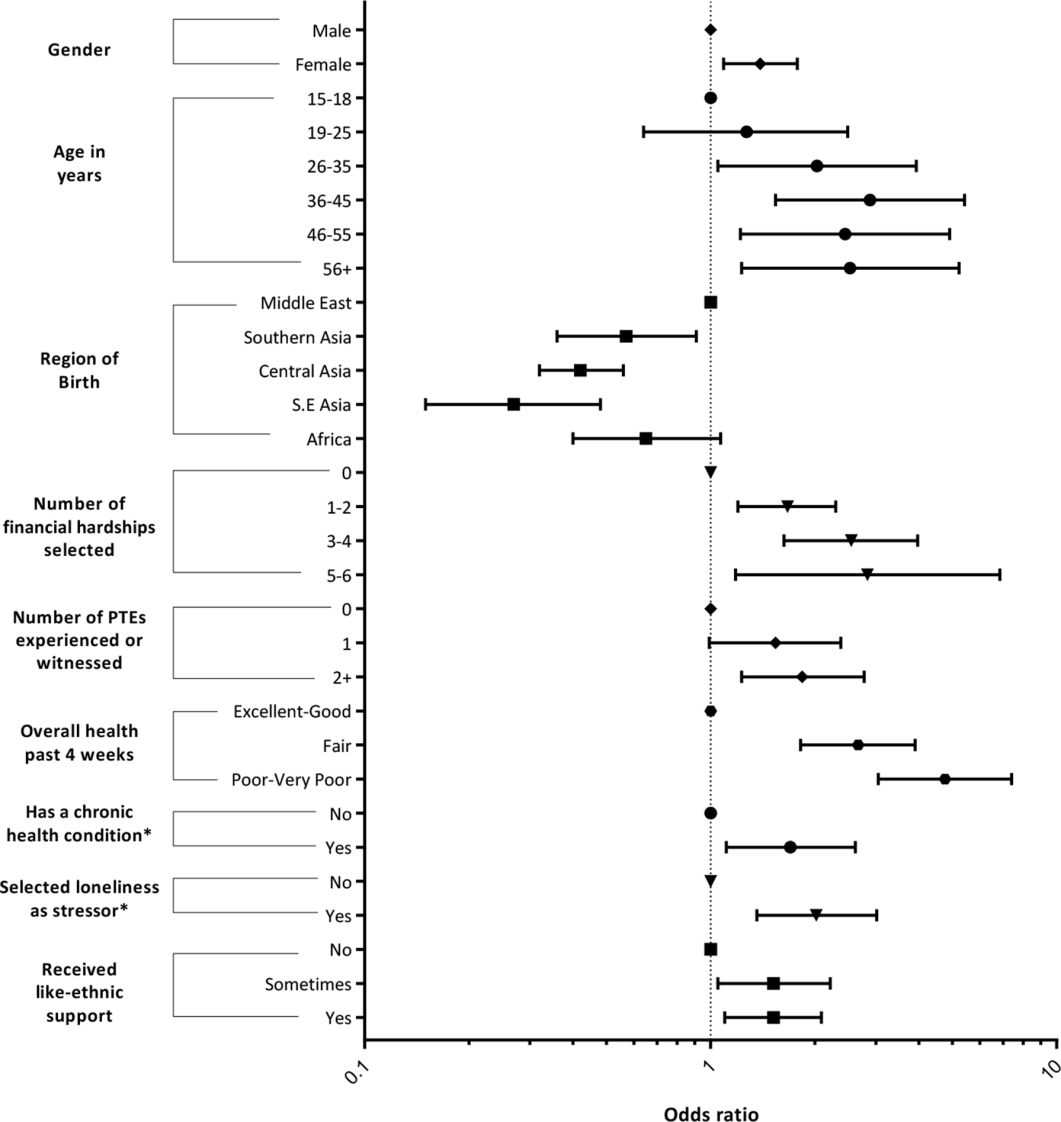
Forest plot of significant predictors from PTSD. Summary of variable selection process and results. “Religion” variable removed at multivariate analysis stage, as it did not achieve significance (p < 0.1) in most categorical responses and the number of participants in certain response categories was low. This meant that the model failed to converge when the “Religion” variable was included and was removed at this stage of analysis.

**Tables 6** and **7** show the results of GLMMs with positive screen for HR-SMI and PTSD as binary outcomes after 200 bootstrap replications. **Figures 3** and **4** represent forest plots of significant results from these final models. Model 1 included all variables arising from the variable selection process. Model 2 was a sensitivity analysis that excluded variables with >10% missing data across all waves. Both models detected the same independent variables reaching significance, which demonstrates robust model outputs. Model 2 reported more significant categories within the independent variables and has smaller AIC and BIC scores. However, model 1 includes variables with >10% missing data across all waves and subsequently maximizes the number of data points used in the model.

**Table 6:** GLMM high risk of severe mental illness. Results from generalized linear mixed models using “**High risk** of severe mental illness” as positive outcome.

		Model 1			Model 2		
Variable	Response	*OR*	*p*	*95% CI*	*OR*	*p*	*95% CI*
**Survey wave**	*1*	–	–	–	–	–	–
	*2*	0.59	0.095	(0.32–1.10)	0.67	0.060	(0.45–1.02)
	*3*	1.09	0.741	(0.65–1.83)	1.09	0.691	(0.72–1.66)
**Gender**	*Male*	–	–	–	–	–	–
	*Female*	1.73	0.012	(1.13–2.64)	1.51	0.010	(1.10–2.08)
**Region of birth**	*Middle East*	–	–	–	–	–	–
	*Central Asia*	0.60	0.024	(0.39–0.94)	0.57	0.003	(0.39–0.83)
	*Southern Asia*	0.47	0.065	(0.21–1.05)	0.29	<0.001	(0.15–0.58)
	*S.E Asia*	0.24	0.114	(0.40–1.41)	0.14	0.002	(0.04–0.49)
	*Africa*	0.53	0.084	(0.26–1.09)	0.51	0.042	(0.27–0.97)
**Housing contract**	*Long term contract (>6 months)*	–	–	–	–	–	–
	*Short term lease (<6 months)*	1.15	0.644	(0.64–2.07)	1.18	0.442	(0.77–1.83)
	*No/temporary contract*	2.10	0.085	(0.90–4.89)	2.31	0.018	(1.16–4.61)
**Number of financial hardships**	*0*	–	–	–	–	–	–
	*1–2*	1.80	0.035	(1.04–3.11)	1.97	0.003	(1.26–3.10)
	*3–4*	3.69	<0.001	(1.83–7.43)	4.00	<0.001	(2.13–7.49)
	*5–6*	2.75	0.119	(0.77–9.81)	3.50	0.011	(1.33–9.18)
**Has a chronic health condition**	*Yes*	–	–	–	*	*	*
	*No*	1.75	0.035	(1.04–2.94)	*	*	*
**Overall health past 4 weeks**	*Excellent–Good*	–	–	–	–	–	–
	*Fair*	2.58	0.002	(1.41–4.71)	3.78	<0.001	(2.40–5.95)
	*Poor–Very Poor*	7.92	<0.001	(3.81–16.46)	13.3	<0.001	(7.38–23.9)
**Discrimination**	*No*	–	–	–	–	–	–
	*Yes*	1.82	0.161	(0.79–4.22)	2.10	0.033	(1.06–4.14)
**Selected loneliness as stressor**	*No*	–	–	–	*	*	*
	*Yes*	2.07	0.007	(1.23–3.49)	*	*	*
**Selected finances as stressor**	*No*	–	–	–	*	*	*
	*Yes*	1.72	0.042	(1.02–2.91)	*	*	*
**AIC Criteria**			3,191			3,632	
**BIC Criteria**			3,327			3,751	
**Number of observations**			4,829			5,563	

OR, odds ratio; p, p-value; CI, confidence interval; AIC, Akaike’s Information Criteria; BIC, Bayesian information criteria; S.E Asia, South-East Asia. – = reference response. * = variable not included in model due to missing data >10%. Model 2 includes variables with missing data >10% from the variable selection process. Model 1 includes all variables from the variable selection process.

**Table 7:** GLMM PTSD. Results from generalized linear mixed models using “PTSD” as positive outcome.

		Model 1			Model 2		
Variable	Response	*OR*	*p*	*95% CI*	*OR*	*p*	*95% CI*
**Survey wave**	*1*	–	–	–	–	–	–
	*2*	0.49	0.002	(0.31–0.77)	0.57	0.001	(0.41–0.79)
	*3*	0.83	0.322	(0.58–1.19)	0.81	0.200	(0.59–1.12)
**Age**	*15–18*	–	–	–	–	–	–
	*19–25*	1.91	0.204	(0.70–5.21)	1.27	0.494	(0.64–2.49)
	*26–35*	2.59	0.044	(1.02–6.54)	2.03	0.034	(1.05–3.93)
	*36–45*	3.57	0.008	(1.89–9.18)	2.89	0.001	(1.54–5.42)
	*46–55*	2.82	0.034	(1.08–7.38)	2.45	0.011	(1.22–4.91)
	*56+*	3.05	0.027	(1.13–8.21)	2.53	0.012	(1.23–5.23)
**Gender**	*Male*	–	–	–	–	–	–
	*Female*	1.41	0.019	(1.06–1.89)	1.39	0.008	(1.09–1.78)
**Region of birth**	*Middle East*	–	–	–	–	–	–
	*Centra Asia*	0.38	<0.001	(0.26–0.56)	0.42	<0.001	(0.32–0.56)
	*Southern Asia*	0.68	0.111	(0.42–1.09)	0.57	0.019	(0.36–0.91)
	*S.E Asia*	0.34	0.003	(0.16–0.70)	0.27	<0.001	(0.15–0.48)
	*Africa*	0.67	0.205	(0.36–1.25)	0.65	0.089	(0.40–1.07)
**Number of PTEs experienced or witnessed**	*0*	–	–	–	–	–	–
	*1*	1.58	0.048	(1.00–2.49)	1.54	0.054	(0.99–2.38)
	*2+*	1.83	0.005	(1.20–2.80)	1.84	0.003	(1.23–2.78)
**Number of financial hardships**	*0*	–	–	–	–	–	–
	*1–2*	1.76	0.005	(1.18–2.62)	1.67	0.002	(1.20–2.30)
	*3–4*	2.74	<0.001	(1.58–4.74)	2.55	<0.001	(1.63–3.97)
	*5–6*	2.52	0.090	(0.87–7.31)	2.84	0.020	(1.18–6.86)
**Has a chronic health condition**	*No*	–	–	–	*	*	*
	*Yes*	1.70	0.015	(1.11–2.62)	*	*	*
**Overall health past 4 weeks**	*Excellent–Good*	–	–	–	–	–	–
	*Fair*	2.51	<0.001	(1.57–4.01)	2.67	<0.001	(1.82–3.90)
	*Poor–Very Poor*	3.53	<0.001	(2.06–6.06)	4.76	<0.001	(3.05–7.42)
**Selected loneliness as stressor**	*No*	–	–	–	*	*	*
	*Yes*	2.02	0.001	(1.36–3.02)	*	*	*
**Received like-ethnic support**	*No*	–	–	–	–	–	–
	*Sometimes*	1.53	0.075	(0.96–2.44)	1.52	0.028	(1.05–2.22)
	*Yes*	1.62	0.027	(1.06–2.48)	1.51	0.011	(1.10–2.09)
**AIC Criteria**			**4,812.0**			**5,634.6**	
**BIC Criteria**			4,980.9			5,794.2	
**Number of observations**			4,894			5,713	

OR, odds ratio; p, p-value; CI, confidence interval; AIC, Akaike’s information criteria; BIC, Bayesian information criteria; S.E Asia, South-East Asia. – = reference response. * = variable not included in model due to missing data >10%. Model 2 includes variables with missing data >10% from the variable selection process. Model 1 includes all variables from the variable selection process.

No social support variables were significantly associated with any study outcomes in multivariate analysis stages (see **Supplementary Appendix**), except like-ethnic support, which was significantly positively associated with PTSD (**Table 7**). Loneliness was significantly positively associated with all study outcomes (**Tables 6** and **7**).

Other variables showing a significant positive association with HR-SMI were female gender, Middle Eastern region of birth, unstable housing contract, increasing financial hardship, increasing poor self-rated health, having a chronic health condition, experiencing discrimination, and financial stress (**Table 6**).

Other variables showing a significant positive association with PTSD were older age, female gender, Middle Eastern region of birth, increasing number of traumatic events, increasing financial hardship, presence of a chronic health condition, and increasing poor self-rated health (**Table 7**).

## Discussion

Our study considers three waves of data from the largest, nationally representative, prospective cohort study of refugees conducted to date (*n* = 2,399). Our study considers data from the largest prospective, population level, cohort study of refugee populations to date. The nationally representative nature of the cohort is rarely seen with research considering refugee populations. Our study is the first to use all three waves of data from the total BNLA cohort. We show the predictors of mental illness in this cohort and examine the relationship of like-ethnic social support and mental illness.

The most important finding from this study is that there was a high prevalence of mental illness that persisted over the first 3 years of resettlement in Australian humanitarian migrant populations. Although there was a significant decrease in prevalence of all primary study outcomes at wave 2, there was no significant decrease of HR-SMI from waves 1 to 3. There was a statistically significant yet perhaps clinically insignificant decrease in prevalence of PTSD from waves 1 to 3. These findings do not support our first hypothesis, and this trough in mental illness prevalence may be due to interview mode effects at wave 2, which mainly consisted of computer-assisted telephone interviews.

Our results agree with the findings from the meta-analysis of Steel et al. who showed prevalence of PTSD as 30.6% (95% CI, 26.3–35.6%) and depression as 30.8% (95% CI, 26.3–35.6%) in refugees and conflict-exposed populations (6). Our results also agree with Hauff and Vaglum, who showed no decline in general psychological distress and depression over the first 3 years of resettlement in a small sample (*n* = 145) of Vietnamese refugees in Norway (18). Our findings diverge from those of other authors who have shown a decrease in prevalence of mental illness as time in host country increased in Australian (17) and Canadian (11) resettlement contexts. As our results are taken from a large, representative, longitudinal sample of the current Australian refugee population, we argue that they hold particular significance for the humanitarian migrant populations resettled both in Australia and worldwide today.

Contrary to our second hypothesis, like-ethnic support showed a positive association with PTSD. We failed to detect statistically significant multivariate associations between other social support variables and all outcome measures during our variable selection process. This is an unexpected finding and diverges from results from past authors of qualitative (12, 13) and quantitative (10) studies, who have shown the beneficial effects of like-ethnic support for mental health. Rarely have authors shown that a greater degree of support from a like-ethnic community is positively associated with mental illness (14). Given that our findings show an associative relationship, it may be that individuals suffering from PTSD symptomatology may be receiving greater degrees of like-ethnic support or may be reluctant to seek external sources of support due to avoidant symptoms typical of PTSD. Alternatively, given that discrimination also emerged as a predictor for PTSD and severe mental illness, it may also be that the positive association of like-ethnic support and mental illness reflects a disenfranchisement of individuals from the wider Australian society. This appears similar to findings from Beiser (29), who showed that the negative effects of discrimination on depression were amplified among those with greater attachment to their ethnic identity. Reminders of premigration trauma upon interaction with members of a like-ethnic community, causing PTSD symptomatology may also be a factor in this unexpected finding. Further research, including qualitative research and subgroup analysis, particularly of adolescents within the cohort, is needed to examine the nature of like-ethnic social support through resettlement in relation to mental health. Future research using trajectory modeling techniques would also allow an examination on individual illness trajectory within the BNLA cohort.

Other significant associations highlight the importance of socioeconomic stability in the post-migration context. Significant associations in this post-migration context represent modifiable factors. The dose–response-like relationship with financial hardship and mental illness appears strong, as was a similar dose–response with premigration trauma and PTSD, and self-rated health and mental illness.

Unemployment was not a significant predictor of mental illness, contrary to past studies (30–32), and noted is the extremely low employment rate even at 3 years (see **Table 2**), making it likely to be underpowered in this study.

Middle Eastern region of birth emerged as a significant predictor of PTSD and HR-SMI (see **Figures 3** and **4**) when compared with other nationalities. This agrees with recent studies showing high prevalence of PTSD and depression among Middle Eastern populations and has clinical significance given the current Middle Eastern refugee crisis (33, 34).

### Limitations

There are limitations to our study and the original design of the BNLA survey. All survey variables were self-reported variables, often based on single-item responses. Our measure of “like-ethnic support” in this way failed to explicitly characterize the nature of the support received and accordingly may have had an array of interpretations, especially across the 17 languages it was translated into. Likewise, the ambiguous wording of “other community groups” when characterizing nonethnic support variables is an issue of study design. This may introduce bias through diverse interpretations of the meaning of questions. There is also a potential for nonresponse bias and attrition bias given that only 1,509 PAs completed baseline interviews out of an eligible 4,035 in the DIBP database. There was also an overall attrition rate of 79%, which we would argue is acceptable for this highly mobile and “hidden” population (21). Although the psychometric tools used in this study show strong internal consistency, false prevalence estimates may occur due to the inadequacy of psychometric tools to detect the full range of culturally variant expressions of mental illness (35). Likewise, when considering final predictors, we considered our population as a homogenous sample, when in fact they are a heterogeneous one, both by ethnicity but also by migration pathway. The onshore and offshore subgroups within our population differed significantly by demographic characteristics and may confound results. Differences in baseline gender, education, employment, and time since arrival may explain the unexpected greater prevalence of mental illness in the onshore subgroup. Our study was also unable to effectively examine the effect of intervention with medication or other evidence-based treatment for mental illness, as questions concerning these factors were not asked at each wave consistently (see **Table 4**).

Given this inherent heterogeneity however, it is likely our results apply to similar resettlement contexts worldwide, as our population is nationally representative of Australia’s contemporaneous refugee intake and the populations currently displaced globally.

### Implications of the Findings

Humanitarian migrant populations remain at a high risk for mental illness over the first 3 years of resettlement. Post-migration variables represent modifiable targets for intervention and highlight the importance of post-migration socioeconomic stability. Given the limitations considered, our results show that refugees who recently resettled in Australia are a cohort at high risk of mental illness given that the majority (52.4%) of the participants screened positive for PTSD or HR-SMI at some point in the study. Persisting high prevalence of mental illness over the first 3 years shows a possible unmet need for trauma-informed mental health service provision to newly arrived humanitarian migrants extending beyond the first year of resettlement.

The results imply that screening for mental illness may be necessary over the first 3 years of resettlement and could be a valuable part of resettlement services. Given that perceived physical health is an important positive predictor, primary care clinics could be an important point of screening. This is also echoed in the previous literature concerning somatization of mental illness in culturally diverse populations.

The results support the importance of specific planning for mental health service provision in areas of high refugee settlement, which may also be areas of socioeconomic disadvantage. Recent examination of national surveys showed socioeconomically disadvantaged areas to have significant effects on prevalence of elevated K10 scores (36). The RR in this examined data for having a K10 score of ≥20 in the most disadvantaged group compared with that in the least disadvantaged group was 2.1 nationally. This finding may reflect the combination of social drift, occupational and environmental adversity, and relative undertreatment in disadvantaged areas (4). The level of distress in the RHS clinic population was more than three times that in the matched Australian-born sample. This was greater than to be expected from socioeconomic disadvantage alone and, therefore, likely causally associated with the high levels of exposure to traumatic events, stressful migration experiences, and the additional barriers to health care such as culture and language experienced by refugees and asylum seekers (4), all of which present additional challenges in providing equitable mental health care.

The positive association between like-ethnic social support and mental illness was an unexpected finding, and further mixed-methods research is needed to help illuminate the association between PTSD and ethnic-like support. At present, the conclusions that can be drawn from it are limited, and as this finding has rarely been documented in the past literature, it raises interesting avenues for future research. Further subgroup analysis is another important avenue of future research for this heterogenous population. This could include qualitative studies among culturally diverse humanitarian migrant populations to enable a greater interpretation of our results and their applicability to guiding evidenced-based interventions.

Finally, other predictors including perceived loneliness arising from our results add to the prior body of evidence highlighting the importance of stability in the post-migration context. The significant post-migration predictors shown in our results represent modifiable factors for intervention by government and mental health service providers.

## Author Contributions

SC: Literature search, figures, study design, data analysis, data interpretation, writing. JE: Study design, data analysis, data interpretation, writing. FS: Study design, data interpretation, writing. GM: Study design, data interpretation, data interpretation, writing.

## Conflict of Interest Statement

The authors declare that the research was conducted in the absence of any commercial or financial relationships that could be construed as a potential conflict of interest.
