# Supplementary material for: Determinants of Mental Illness Among Humanitarian Migrants: Longitudinal Analysis of Findings From the First Three Waves of a Large Cohort Study
Source: Front Psychiatry. 2019 Aug 2;10:545. doi: 10.3389/fpsyt.2019.00545 (PMC6688655; doi:10.3389/fpsyt.2019.00545)
Supplement: Supplementary file 1 [file DataSheet_1.docx]

# Supplementary Appendices

## GLMM models

**BNLA Panel**

**GLMM Level & Model**

*Level 1*

Random effects model

*Level 2*

Random effects model

**W1**

**W2**

**W3**

Repeated measures within individuals
(annual waves)

*Level 3*

Fixed effects model

Migrating units (families)

**PA**

**SA**

Between individuals

**Figure S1:** Summary of generalised linear mixed models applied to the BNLA data set. Author’s own figure adapted from previous unpublished diagram from supervisor (J.E). PA =Principal applicant. SA=Secondary applicant. W1=Wave 1. W2=Wave 2. W3=Wave 3. GLMM=Generalised linear mixed model. BNLA=Building a New Life in Australia study.

## Binomial tests

|  |  | Waves | | |
| --- | --- | --- | --- | --- |
|  |  | *1→2* | *2→3* | *1→3* |
| *Two sided p value* | *PTSD* | <0.001ᶧ | 0.009ᶧ | 0.043ᶧ |
|  | *HR-SMI* | 0.015ᶧ | 0.005ᶧ | 0.775 |
| *One sided p value* | *PTSD* | <0.001* | 0.005** | 0.021* |
|  | *HR-SMI* | 0.007* | 0.003** | 0.620 |

**Table S1:** Results from binomial probability tests for all participants. Author’s own. ᶧ indicates significant difference in prevalence between waves. *indicates significant decrease in prevalence between waves. ** indicates significant increase in prevalence between waves. Significance level = p<0.05. PTSD=Post-traumatic stress disorder. HR-SMI=High risk severe mental illness

## Results from variable selection process

'Gender'

'Region of birth'

'Housing tenure'

'Financial hardships'

'Chronic health condition'

**Univariate Analysis**

**Collinearity**

**Multivariate Analysis**

**Bootstrapping**

**Final Variables**

**37 Candidate Variables identified from dataset**

**9**

**17**

**28**

**34**

*Univariate ordinal regression*

*Pearson’s correlation coefficient*

*Multivariate panel data logit models*

*(Population averaged)*

*Logit models bootstrapped*

Using baseline variables and ordinal primary and secondary outcomes for all 3 waves. Variables retained at p < 0.1

***Variables Excluded:***

‘Remoteness’

Examined correlation between variables. One candidate variable selection from two or more collinear variables when r > 0.5

1. Variables retained at p < 0.1 for primary and secondary outcomes.

2. Social support and time variables retained only at p < 0.1

**10**

**11**

***Variables Excluded:***

‘Time on bridging visa’

'Time in immigration detention'

'Migration pathway'

'Spoken English understanding'

'English writing proficiency'

'English reading proficiency'

***Variables Excluded:***

*‘Religion’***

*'Married/partnered'*

*'SEIFA scale of disadvantage'*

*'Spent time in Refugee camp'*

*'Current English speaking proficiency' 'Stress: Language barrier'*

*'Stress: work'*

*'Currently employed'*

*'Family waiting to come to Australia'*

*'Support: Religious'*

*'No social support'*

*'Time since arrival'*

***Variables Excluded:***

***Severe mental illness***

'Trauma'

***PTSD***

'Degree of social support'

'Migrating Unit Structure'

'# times moved since last interview'

'Pre-arrival education'

'Support from other community'

'Discrimination'

***Any mental illness***

'Migrating unit structure'

'# times moved since last interview'

Variables retained if p < 0.05 in ≥ 50% of bootstrap samples

*HR-SMI*

'Age'

'Gender'

'Region of birth'

'Trauma'

'Financial hardships'

'Chronic health condition'

'4 week physical health'

'Stress: Loneliness'

'Like-ethnic support'

'4 week physical health'

'Discrimination'

'Stress: Loneliness'

'Stress: Finances'

'Age'

'Gender'

'Region of birth'

'Trauma'

'Financial hardships'

'Chronic health condition'

*PTSD*

*Any mental illness*

'4 week physical health'

'Stress: Loneliness'

'Like-ethnic support'

**Figure S2:** Summary of variable selection process and results**.** Author’s own figure. ****** ’Religion’ variable removed at multivariate analysis stage as it did not achieve significance (p < 0.1) in most categorical responses and the number of participants in certain response categories was too low. This meant that the model failed to converge when the ‘Religion’ variable was included and was removed at this stage of analysis.

## Key study variables considered in final models

|  |  |  | Present in final GLMM | |
| --- | --- | --- | --- | --- |
| Variable | **Categories** | **Contents of variable** | ***PTSD*** | ***Severe mental Illness*** |
| Wave | *1, 2, 3* | Represents each survey wave. Wave 1 = baseline | ✓ | ✓ |
| Age | *15-18,19-25, 26-35, 36-45, 46-55, 56+* | Age in integer years, derived from DIBP database | ✓ |  |
| Gender | *Male, Female* | Male and female binary derived from DIBP | ✓ | ✓ |
| Region of birth | *Middle East, Central Asia, Southern Asia, S.E Asia, Africa* | Major groups based on the Standard Australian Classification of Countries minor groups. ‘North Africa’, ‘Central and West Africa’, ‘Southern and East Africa’ recoded as ‘Africa’. ‘Mainland South-East Asia’ and ‘Maritime South-East Asia’ recoded as ‘South-East Asia’. | ✓ | ✓ |
| Education level | *Never attended school, 6 or less years of school, 6-12 years of school, Trade/Technical qualification, University degree* | Self-reported highest level of education attained prior to arrival in Australia.  Question asked: *What is the highest level of education you completed before coming to Australia?* |  |  |
| Number of potentially traumatising events (PTE) experienced or witnessed | *0, 1, 2+* | Aggregate of 7 separate variables, each received a score of 1 for each of the below traumas selected. These were then added and a total score was recoded into appropriate categories. Question asked:  *Please select from the following list the events you have experienced or witnessed:*   - *Extreme living conditions (e.g. lack of food, water, shelter or medicine)* - *Combat exposure* - *Violence* - *Imprisonment/kidnapping* - *Political/religious persecution* - *Natural disasters* - *Other trauma* | ✓ |  |
| Number of financial hardships | *0, 1-2, 3-4, 5-6* | Aggregate of 6 separate variables based on PA answer. Each financial hardship received a score of 1 if selected, scores were then summer and recoded into appropriate categories. Question asked:  *In the last 12 months, has any of the following happened to you because you didn't have enough money?*   - *Could not pay gas, electricity or telephone bills on time* - *Could not pay the rent or mortgages on time* - *Went without meals* - *Were unable to heat or cool your home* - *Pawned or sold something because you needed cash* - *Needed help from a welfare or community organisation* | ✓ | ✓ |
| Has a chronic health condition | *Yes, No* | Self-reported binary response variable. Question asked:  *Do you have a disability, injury or health condition that has lasted or is likely to last 12 months or more?* | ✓ | ✓ |
| Overall health past 4 weeks | *Excellent-Good, Fair, Poor-Very Poor* | Self-rated health scale recoded into condensed categories. Question asked:  *Overall, how would you rate your health during the past 4 weeks?*   - *Excellent* - *Very good* - *Good* - *Fair* - *Poor* - *Very Poor* | ✓ | ✓ |
| Discrimination | *Yes, No* | Self-reported binary response variable. Question asked:  *In the last 12 months, have you been discriminated against, stopped from doing something, or been hassled or made to feel inferior, because of your ethnicity, religion or skin colour?*   - *Yes* - *No* |  | ✓ |
| Selected loneliness as stressor | *Yes, No* | Self-reported binary response variable. Question asked:  *Is the following a source of stress in your life?*   - *Loneliness* | ✓ | ✓ |
| Received like-ethnic support | *Yes, Sometimes, No* | Self-reported categorical response variable. Question asked:  *Do you feel that you have been given support/comfort in Australia from your national or ethnic community in the past 12 months?*  *1. Yes. 2. Sometimes. 3. No* | ✓ |  |
| Received religious support | *Yes, Sometime, No* | Self-reported categorical response variable. Question asked:  *Do you feel that you have been given support/comfort in Australia from your religious community in the past 12 months?*  *1. Yes. 2. Sometimes. 3. No* |  |  |
| Received other support | *Yes, Sometimes, No* | Self-reported categorical response variable. Question asked:  *Do you feel that you have been given support/comfort in Australia from other community groups in the past 12 months?*  *1. Yes. 2. Sometimes. 3. No* |  |  |
| Degree of support | *High, Low* | Aggregate variable created from addition of 3 social support variables: ‘Ethnic’ + ‘Religious’ + ‘Other’. Yes received score of 2, Sometimes received score of 1, No Received score of 0.  All aggregate scores >2 recoded as ‘High’. All aggregate scores ≤2 recoded as ‘Low’ |  |  |
| Received no Support | *Yes, No* | Aggregate variable. If respondent selected ‘No’ at each of *‘Ethnic*‘, ‘*Religious’* & *‘Other support’* questions the respondent received a score of ‘Yes’ at each given wave. |  |  |
| Housing contract | *Temporary, Short term (<6 months), Long term (>6 months)* | Categorical response variable, other recoded as missing due to low response (<1.7%). Question asked:  *Is your current housing arrangement...?*   - *Temporary* - *Short term (<6 months)* - *Long term (>6 months)* - *Other* |  | ✓ |

**Table S2. Key study variables considered in final models.** The table also shows the variables included in the final models.

|  |  |
| --- | --- |
| Variable | **Response** |
| Age | *14-18* |
|  | *19-25* |
|  | *26-35* |
|  | *36-45* |
|  | *46-55* |
|  | *56+* |
| Gender | *Male* |
|  | *Female* |
| Region of birth | *Middle East* |
|  | *Central Asia* |
|  | *Southern Asia* |
|  | *S.E Asia* |
|  | *Africa* |
| Religion | *Buddhism* |
|  | *Christianity* |
|  | *Hinduism* |
|  | *Islam* |
|  | *Other* |
|  | *No religion* |
| Married or partnered | *No* |
|  | *Yes* |
| SEIFA decile of disadvantage | *1-5* |
|  | *6-10* |
| Remoteness | *Metropolitan* |
|  | *Inner regional* |
|  | *Outer regional* |
| MU structure | *Couples* |
|  | *Single parent* |
|  | *Single with other family members* |
|  | *Single individual* |
| Education level | *Never attended school* |
|  | *6 or less years of school* |
|  | *6-12 years of school* |
|  | *Trade/technical qualification* |
|  | *University degree* |
| Migration pathway | *Onshore* |
|  | *Offshore* |
| Time since arrival | *<1 year* |
|  | *1-2 years* |
|  | *2-3 years* |
|  | *3+ years* |
| Number Potentially Traumatic Events experienced or witnessed | *0* |
|  | *1* |
|  | *2+* |
| Spent time in Immigration Detention | *Yes* |
|  | *No* |
| Spent time in Community Detention | *Yes* |
|  | *No* |
| Spent time on Bridging Visa | *Yes* |
|  | *No* |
| Spent time in refugee camp | *No* |
|  | *Yes* |
| Currently understands spoken English | *Very well/Well* |
|  | *Not well/Not at all* |
| Current English speaking proficiency | *Very well/Well* |
|  | *Not well/Not at all* |
| Current English reading proficiency | *Very well/Well* |
|  | *Not well/Not at all* |
| Current English writing proficiency | *Very well/Well* |
|  | *Not well/Not at all* |
| Housing contract | *No contract/Temporary* |
|  | *Short term lease (<6 months)* |
|  | *Long term contract (>6 months)* |
| Number of times moved since arriving in Australia | *0* |
|  | *1* |
|  | *2+* |
| Currently employed | *Yes* |
|  | *No* |
| Has family waiting to come to Australia | *Yes* |
|  | *No* |
| Number of financial hardships | *0* |
|  | *1-2* |
|  | *3-4* |
|  | *5-6* |
| Degree of social support | *Low* |
|  | *High* |
| No support | *No* |
|  | *Yes* |
| Received support from like-ethnic community | *Yes* |
|  | *Sometimes* |
|  | *No* |
| Received support from religious community | *Yes* |
|  | *Sometimes* |
|  | *No* |
| Received support from other community | *Yes* |
|  | *Sometimes* |
|  | *No* |
| Has chronic health condition | *Yes* |
|  | *No* |
| Overall health past 4 weeks | *Excellent-Good* |
|  | *Fair* |
|  | *Poor-Very Poor* |
| Discrimination | *Yes* |
|  | *No* |
| Selected loneliness as stressor | *No* |
|  | *Yes* |
| Selected finances as stressor | *No* |
|  | *Yes* |
| Selected language barriers as stressor | *No* |
|  | *Yes* |
| Selected work as stressor | *No* |
|  | *Yes* |

**Table S3. List of all candidate variables considered for stage 1 of variable selection process**
